# Address to the Graduates

**Published:** 1884-05

**Authors:** Frank A. Hunter

**Affiliations:** Cincinnati, O.


### ADDRESS TO THE GRADUATES.

BY FRANK A. HUNTER, D.D.S., CINCINNATI, O.

[At the Ohio College of Dental Surgery, March 4th, 1884.]

*Gentlemen of the Graduating Class:*

Having been a graduate myself, and having occupied the same position that you do to-night, I can very well appreciate your feelings, when the thought comes to you that I, through the invitation of your Faculty, and in a position to inflict upon you, torture, to which the agonies of the Spanish Inquisition were mild indeed.

The poet has informed us that revenge is sweet; and it is with pleasure that I accept the task of making you as miserable as was I upon a similar occasion.

> "Breathes there the man with soul so dead
> Who never to himself hath said
> My time will come?"

Gentlemen, it has come—I am here.

Following the custom of my predecessors, I have arranged my address under twenty-three separate headings:

First, considerable time and space are usually employed by speakers and writers to demonstrate that the practice of dentistry is an honorable calling; I will simply dismiss so self-evident a proposition by saying that honestly conducted, our profession is inferior to none.

The antiquity of dentistry is and has been a favorite theme with many writers; and it has been traced to different periods of the world's history. It has been claimed that recently, by the unwrapping of Egyptian mummies, gold fillings have been discovered in the teeth. Some people are skeptical enough to suggest that probably these fillings have been put there after the mummy was unwrapped. Be that as it may, I have seen conclusive evidence that dentistry was known, and that the teeth were cared for, by pre-historic man. In the Nevada silver mines, while wandering along one of the drifts or galleries, many hundreds of feet below the surface of the earth, where all is darkness save for the torch that illuminates the way, I suddenly came upon a huge wall of stone, containing what to me was evidence of the great antiquity of our profession; for there the miners, by their excavations, had disclosed, written upon the stone in symbols which it was impossible to misconstrue, "Sozodont; for the teeth."

Still following the custom of similar occasions—for gentlemen, we must follow custom; the laws of custom are rigidly drawn, and he who has the hardihood to o'er-step the bounds, is guilty of innovation, and risks his reputation for good taste, and reverence for the past. So following the custom, it becomes my duty to impress firmly upon your minds, the all-important fact that this evening is the commencement of your professional career. Let not your conceit carry you beyond your judgment, and lead you to think that your student life is ended; for, as a rule, he succeeds best whose student life ends only with death; and he who at old age, is satisfied with the knowledge of youth, has been left so far behind in the progress of the world, that he is not thought worthy of confidence by those around him.

In selecting a field wherein to practice your chosen profession, let me advise you to establish yourselves where you are not known; not that I wish to cast any reflection upon your integrity or ability (for graduation under the Faculty of the Ohio Dental College is a synonym for both); but you will secure greater and more speedy success among strangers than you will in your native place, for the simple reason that there you will be looked upon as a boy until you turn gray, and then you will be considered too old to practice.

If your desires or inclinations so lead you, if you feel the need of reliance upon some greater power for your guidance in this world or your salvation in the next, connect yourself with some religious organization; but for God's sake, as well as your own, do not join church for the benefit of business. It is one of the contemptible forms of advertising of which you could be guilty. There is no class of men that more need the saving grace of religion than do the dentists—at least so our patients say—but if there is not a nice pleasant corner in the next world set apart for our exclusive use, there is no such thing as retributive justice.

There are many different ways of working up a dental practice; but the easiest, speediest, most certain and satisfactory, is by advertising—not in the public prints, by hand bills, or by joining church, but by advertising the fact, unostentatiously, that you are a gentleman, and by letting your work advertise your ability.

It would be almost useless for me to conjecture the motives which led you to adopt dentistry as your profession, but I think that I am safe in saying, that the majority of you did so because it seemed to you the most congenial mode of earning a livelihood. Some enter the profession and continue in it, from a sense of duty; for the love of doing good; from purely philanthropic motives. I have known some such, and have universally found their distinguishing characteristic to be exorbitant fees. Others enter the profession because it is an easy way to get rich. All dentists *are* rich, at least they ought to be; for, from the time they commence practice fortune favors them; and it is a difficult matter to find one who did not do a business of over three thousand dollars the first year, forty-five hundred the second, seven thousand the third, and so on, increasing in proportion, so that by the time he had been in practice twenty years, he was able to buy a house and lot *on credit.* Professional men, as a class, live well but die poor. It has been estimated that in an experience of twenty-five years in a mercantile pursuit, the percentage of failures is about ninety-seven in the hundred; and where the failure occurs after middle life, and is honest, the merchant loses his capital, and is seldom able to get upon his feet again. But with the professional man, his health is his capital; if he should get a little ahead, and desire to speculate, and stake his pile on an ace full, against four jacks, he loses his money; but still, having his health, he is in position to call for a new deal, and take a fresh start.

There is not the same chance in a professional career for making a large amount of money as in mercantile pursuits, neither is there the same chance to lose what you have already made. It is a more regular occupation, and if you behave yourself properly you can make a comfortable living.

You now start out with an unlimited fund of knowledge—in your mind. As you grow, it will grow—beautifully less; and at the end of twenty-five years, if you progress as I hope you will, you will have come to the conclusion that you don't know anything. That may seem paradoxical to you, but it is true nevertheless.

Associate yourselves with one or more of our Dental Societies.

No one man knows it all, and by the mutual interchange of ideas great good is accomplished. The social aspect alone is worth the time and trouble of attending the meetings, for it is pleasant to make acquaintances among men in the same occupation as ourselves; and during the discussions of the various topics, something *may* possibly be said that will be of benefit to you. Subscribe for one or more of our best dental journals; study them carefully during your *leisure* hours, and if you should find an article that seems to you good, why, read it again. Be contributors to dental science. If you have the talent, write for the journals, but be sure that you have something to say. Do not write simply for the sake of seeing your name in print; and if your line of thought should lead you to *discover* something that was used and discarded before you entered the profession, do not be discouraged, others have done the same thing, but wipe off your pen and try it again.

Besides the studies relating strictly to our profession, it is well to take up some congenial outside study, so to speak, that it may be a recreation to you in after years when engaged in full practice. To a dentist in full practice, the nervous strain is considerable, and the evening study of some congenial theme is a relief. You might take up zoology, geology, botany, astronomy (astronomy is a good *night* study), or microscopy. Now the use of the microscope is entertaining as well as instructive, and most of you being young, it is well enough for you to commence at once, for I believe they say that it takes about one hundred and fifty years constant study to be able to distinguish the difference between the leptothrix buccalis, and the micrococcus.

In working up a successful dental practice, more is required than simple ability to do good work. Tact, management, industry, neatness, sobriety, and honesty, play important parts in the lives of successful practitioners. I have known men of ability who could be classed as eminently successful failures, through lacking one or more of the attributes mentioned. During your career you will undoubtedly find abundant opportunity for the exercise of charity. It remains with yourselves whether you will accede to one of the noblest impulses of mankind. Patients will apply to you for services who are unable to recompense you proportionately to the time and labor expended. Knowing such to be the case, and undertaking the operation, do not slight your work, but render the best service in your power. Neither on the the other hand, increase your fees to those who are in better circumstances, thereby making others pay for your charity. That is not only uncharitable but down right dishonesty.

In your office as well as elsewhere, let your manner be agreeable. You will be the gainers by it. In your work you will find much that will be irritating—so will your patients—but no matter what transpires, keep an unruffled exterior, however great the fire may be raging within.

Furnish your office neatly but not gaudily. Do not go into debt unless you can see your way out. You must creep before you can walk; and from time to time, as your means permit, you can make such additions as will be in good taste. Have things pleasant around you; for *you* yourself will be unpleasant enough to your patients.

Educate your patients in your office, but do not talk shop outside. The man who talks of his own affairs, or of himself, is voted a bore in society, and his company, to say the least, is not sought.

Set apart certain hours for labor, and during those hours be found in your office, for it may be possible some one will call who requires your services, or who may want to pay a bill, and it would be mortifying in the extreme not to find you there. If you fail to make a success of dentistry, do not tack on to it the manufacture of ax-handles, but withdraw from the profession and turn your energies to something for which you are more fitted. In your practice you will undoubtedly come across cases somewhat out of the ordinary run, that will baffle your judgment and defy your ability to carry the treatment to a successful termination. In such cases do not rush in blindly and treat haphazard, but study the case carefully; and if unable to treat intelligently, *consult* with some other dentist—not necessarily an older man, but one in whom you have confidence. In the practice of medicine, physicians are in the habit of consulting with each other in serious cases, but with dentists the custom is " more honored in the breach than the observance." There is far too little consultation in the dental profession for our own good ; we are individually too much wrapped up in ourselves, apparently fearful that our patients will think that somebody else knows as much as we do. No physician was ever the loser by consulting with a reputable practitioner, and there is no dentist that will not be respected for it.

Have respect for medicine and its practitioners, but do not confound the two professions of medicine and dentistry. Dentistry is not, never was, and never can be a specialty of medicine. It is a separate and distinct profession, having its own colleges and its own literature.

A knowledge of anatomy, physiology, chemistry and therapeutics is essential to the successful practice of both professions; and these are thought with equal thoroughness in the colleges of both—frequently by the same professors.

Thus far the two professionals travel side by side, and then diverge; the medical practitioner takes up general medicine, while the dentist begins his art.

The art in medicine or surgery, if you please, is so inconsiderable as hardly to be taken into account, while in dentistry it plays so important a part, that he who is without it, is considered unworthy to enter upon its practice; in fact, it is made the test of ability.

Fortunately for the dignity of the profession, but a few clamour for the recognition of dentistry as a specialty of medicine. The vast majority are satisfied with the recognition we get, and that is as doctors of dental surgery.

It is not an infrequent occurrence for dentists to be called in consultation with medical practitioners, and it is when the latter recognize the case to be outside the science of medicine, and within the science or art of dentistry.

I would not be understood as saying that a medical education is not advisable in the practice of dentistry, but simply that it is not essential. A thorough understanding of the studies taught, in our own colleges, is sufficient to make you successful practitioners.

A knowledge of the natural sciences, and even of law, might sometimes be made available.

Educate your patients not to expect too much of dentistry; and do not expect too much yourselves. One of the greatest drawbacks to the advancement of our profession is the unfulfilled promises of its practitioners. The people at large, through misinformation, are led to expect more from the dentist than they really get. Many think that a tooth once operated upon by the dentist should be as good if not better than it was originally, forgetful of the fact that the work of the Almighty, or Nature, if you please, has failed, before they apply to the dentist for *repairs*. Why should more be expected of man than of Nature? What is to prevent a recurrence of the disease, if the surrounding conditions are not altered? And how seldom can these conditions be changed.

The mere fact that a tooth requires the services of the dentist shows that it is of imperfect structure, or the conditions are favorable to the development of disease. After the diseased portion has been removed and its place supplied by art, it is the duty of the dentist not to "warrant it for life," but to firmly impress upon the mind of the patient the fact that unless more attention is given to it in the future than has been bestowed in the past failure will be the result, and that in a comparatively short time.

We must have the cooperation of our patients, and the sooner that is understood the better it will be for the profession. Some may say why this does not apply to me, for I make no promises at all. Gentlemen, we all make promises. The fact that we operate upon a tooth at all and charge a fee for it is a promise that something is expected; and if the patient expects more than he gets it is the fault of the dentist. I speak thus strongly to show you that the practice of dentistry is not all plain and smooth sailing; that there are obstacles in the way, and that there is a degree of uncertainty about it, as there is in medicine or law.

An incalculable amount of good has been done, and is being done by dental operations; and I am proud to say that I am a member of a profession whose benefits to suffering humanity place it along side of medicine, and whose members take rank with the scientific and thinking men of the world.

But the vast majority of our operations are performed upon teeth of poor structure; and, when we build a house upon a sandy foundation, we must take the chances of a flood, which, coming sooner or later, washes the foundation away and leaves an utter ruin.

And now I have a serious and solemn duty to perform. Take the advice of one whose life has not been all it should have been, one who has lived to see the folly of his way—get married. The benefits to be obtained by such a course are numerous. You will at once secure a large and lucrative practice—among your wife's relations. Be kind and gentle with them; you will be abused, but no matter; let the consciousness of having done a good action uphold you, and you will receive your reward—if not in this world in the next.

In conclusion, allow me to say, let your lives be such that you will obtain the good will and confidence of the community in which you reside. So that when your time has come, and you may have left this world to take your place among the followers of Truth, it may be said of you:

> "His life was gentle; and the elements
> So mixed in him, that Nature might stand up,
> And say to all the world, '*That was a man.*'"

